# Peer Review of “Impact of the COVID-19 Pandemic on Latino Families With Alzheimer Disease and Related Dementias: Qualitative Interviews With Family Caregivers and Primary Care Providers”

**DOI:** 10.2196/56444

**Published:** 2024-03-08

**Authors:** Colleen Peterson

**Affiliations:** 1 Transportation Research Institute (UMTRI), University of Michigan, Ann Arbor, MI, United States

**Keywords:** Latino, dementia, caregiving, COVID-19, Alzheimer’s disease, health disparity, qualitative research, transcript analysis, health inequality, minority population, epidemiology, peer support, social services, health care, Alzheimer’s, minority, qualitative, interview, caregiver, primary care, impact, resilience, disparity, outcome, Alzheimer disease, Alzheimer

*This is the peer-review report for “Impact of the COVID-19 Pandemic on Latino Families With Alzheimer Disease and Related Dementias: Qualitative Interviews With Family Caregivers and Primary Care Providers.”*

## Round 1 Review

### General Comments

The authors [1] present a compelling argument for understanding how a doubly vulnerable population in the United States (Latino persons with dementia) experienced the COVID-19 pandemic. To this end, they share the results of a thematic analysis of interviews with primary care providers and caregivers of Latino persons living with dementia. The qualitative analysis could use better explanations and the themes could be more descriptive. Moreover, the many of themes do not capture or explain their relevance to understanding the intersectionality of dementia and Latino lives. My comments below speak to that, as well as other issues. The manuscript has a good foundation of an informative article that showcases the lived experience of this population during a critical time and could be modified to provide formative evidence for improving care, inside and outside of a pandemic.

### Specific Comments

#### Major Comments

1. Consider making subthemes or codes more descriptive and meaningful. Good themes tell you what the story is or what the direction is at least (good or bad). Some of these are readily available (or easily modified) from sentences in the paper already (eg, “the pandemic influence[d] mental and emotional health”; “Social support was critical for reducing social isolation and its sequalae”; caregivers and persons with dementia “lost access to engaging activities during the confinement”; and “Remote communication facilitated social support”). See other qualitative research for examples. There are even good examples in the dementia care literature during the pandemic. For example:

Mitchell LL, Horn B, Stabler H, et al. Caring for a relative with dementia in long-term care during the COVID-19 pandemic: a prospective longitudinal study. Innov Aging. 2023 Apr 17;7(4):igad034. doi: 10.1093/geroni/igad034. PMID: 37213326; PMCID: PMC10195573.

Harding E, Rossi-Harries S, Gerritzen EV, et al. “I felt like I had been put on the shelf and forgotten about” – lasting lessons about the impact of COVID-19 on people affected by rarer dementias. BMC Geriatr. 2023;23(392). https://doi.org/10.1186/S12877-023-03992-1

2. Throughout: The topic is about the Latino Alzheimer disease and related dementias (ADRD) experience, but many themes do not tap into this overlap of the Latino and ADRD experience. It is not made relevant to the ADRD experience or it is not explained how the ADRD context affected it, either in the theme itself (eg, see “poor nutrition” codes) or in the quote used to justify it (eg, see “stress” and “work” codes). A thorough review of the codes and quotes to meet this intersectionality would be beneficial.

3. Facilitators and barriers are not often used as codes on their own but a way to categorize or further delve into aspects of other issues. What was facilitated? What was the barrier blocking? I could see weaving facilitators, barriers, and consequences into the discussions around the other codes, like informal and formal support.

4. Please clarify the methods.

- I have not heard of using condensed transcripts before. Why was this done? What was taken out exactly? How were “meaningful bits of text” identified?
- It is not clear who was doing the coding at which times. There is the first author as a coder and then 2 additional coders, but the final sentence indicates there were just 2.
- Did the first author make all the themes and do all the coding and then the other coder(s) just reviewed it? (Rather than all independently reviewing and coming together to come up with themes and then independently coding and later addressing the coding discrepancies.)

5. Add headers in the text for each subtheme and the codes for better flow and to help readers keep track of what theme or code they are reading about.

6. For quotes in general:

- Consider editing any quote over 2 lines or selecting briefer quotes. Three lines is okay if compelling. If more than 3, it has to be a really good quote. There are 2 very long quotes in “other impacts.”
- Provide some context or active linking to the code as lead-in text.
- Make sure they are absolutely relevant to the Latino and ADRD experience.

7. For the discussion:

- Consider adding comparisons of findings to non-Latino ADRD COVID-19 experiences to highlight the differences this population experienced and to better showcase why continued attention to this specific population is warranted. See the Mitchell et al and Harding et al papers cited above as potential comparison points.
- Why is circular migration being brought up? As written, this does not appear to be ADRD related and was only lightly discussed in the results (though, it was not clear if it is ADRD related in the results either).
- “The fact that some PCPs suspected an unknown longer-term impact of the COVID-19 pandemic warrants further longitudinal research into this topic.” While true, it is strange to frame ongoing need to understand this based on participant responses alone. Also, it needs to be related to ADRD.
- “Caregivers’ reports on health care providers’ confusion between ADRD and COVID-19 infection symptoms warrants research to improve diagnosis and severity assessments of both conditions.” Where did this come from? It feels unsupported from the evidence provided in the current study and an odd place to end the discussion.

8. For the conclusion:

- “This pandemic has revealed many of the barriers that Latino families with ADRD face, and in most cases, this has exacerbated previous barriers. However, with every crisis comes an opportunity for improvement, which will hopefully translate into improved conditions among Latino families with ADRD.” This does not really say anything; be specific regarding the barriers and what could be improved. You could succinctly use the start of the next sentence to end this one.
- “These improved conditions might include more equitable access to health care and community services, a better quality of these services, subsidized formal and informal supports, and flexible hybrid means of communication.” Which would lead to…or mean what for the (public) health of Latino persons living with ADRD and their carers? What is the overall takeaway pertaining to health or public health?
- The discussion and conclusions could be broadened out to medical care in general if the issues appear to also be independent of the pandemic.

#### Minor Comments

9. Mention the United States as the target population in the abstract.

10. Typos: “The fist author also condensed” and “work in the meat packing plan industry.”

11. Clarify “To make bring rigor and validity.” Or is there a typo here?

12. “To make bring rigor and validity to the research process, the interviewer used active listening techniques during the interview aimed at confirming the information shared by participants. The interviewer also emphasized the fact that participants were the experts in their experiences to reduce power differentials.” This belongs in the methods, not analysis.

13. “[Explains how after the lockdown, the care recipient only remembers long term memories]. So, I was thinking that all the time she was locked down here because of the cold weather and COVID might have affected her more.” Avoid total paraphrasing and provide (translated) direct quotes. Or put the paraphrase as context before the quote.

14. “...PCPs had to reduce physical contact with care recipients, which reduced their chance to convey warmth to their patients.” A quote here would be nice.

15. “This fear was not unfounded. Prior to the availability of the vaccine, caregivers and care recipients acquired COVID-19. As Latino older adults, they were at an increased risk for complications including death, causing significant chronic concern and fear.” This is useful for the introduction (and the vulnerability was discussed) and discussion but should not be a part of the results. I suggest omitting this.

16. “Fourth, care recipients and PCPs highlighted the frequency and severity of depressed mood among caregivers and care recipients, especially during the lockdown due to lack of social support and social isolation. The PCPs noted that lack of social support and social isolation due to lockdown negatively impacted mood, sharing:” This is redundant. Consider condensing into 1 sentence.

17. “Fourth, consequences of social support were physical, psychological, and social. Examples of physical consequences include potentially reducing mortality by providing formal and informal caregiving services. Psychological consequences include clinic and family support reducing loneliness and increasing feelings of safety. Social consequences include caregivers being allowed to accompany their care recipients during clinic visits, curbside visits allowing socialization and home care services lowering isolation.” Like the barriers above this section, these feel more like they could be part of the informal or formal support codes. What are the supporting quotes? Also, it is not clear what were the consequences? What were the causes that led to the consequences?

18. “Third, consequences of the higher use of remote communication were both positive and negative.” This should just be part of the remote communication theme description.

19. “...similar to other studies, the need to rely on remote communication intensified the digital divide.” Reverse it—the digital divide was problematic given the need to rely on remote communication.

20. “...for their survival” in the conclusion is a bit heavy-handed. Speak on something closer at hand in the manuscript like avoiding exposure and infection.

## Round 2 Review

### General Comments

The authors thoroughly attended to the reviewer responses. The methods are easy to understand and the rework of the themes and related quotes in the results is a great improvement. The discussion could be easier to read by breaking the large paragraphs into smaller ones. The conclusion should be revised to more specifically attend to what the study found and how it extends the literature. These and other issues are further noted:

### Specific Comments

#### Major Comments

Major comments in order of appearance in the manuscript:

1. In the 2.2. poor nutrition theme, it is more obvious that the mailing system and financial insecurity could directly be influenced by the pandemic, but it is not clear that skills and level of impairment were affected by the pandemic. As written, it sounds more like an overarching ADRD problem rather than an ADRD issue specific to the pandemic. This should be clarified, especially since it is brought up specifically in the discussion as a unique finding.
2. Consider reworking this sentence to clarify and streamline: “While home-delivered meals operated normally, Latino families with ADRD tried to access these for the first time during the pandemic to obtain food while reducing the risk of infection.” My suggested rework is “Some Latino families with ADRD we interviewed tried to use home-delivered meals for the first time during the pandemic to reduce risk of infection.”
3. Break large paragraphs throughout the discussion into smaller ones by more specific topic (eg, food and nutrition, work changes, and infection risk).
4. How exactly are fatalism and personalism related to the findings in the study? Make an explicit tie back into the findings to make a stronger ending to this part of the discussion.
5. Rephrase “Was this also the case for cognitive and functional decline?” into a statement rather than a question.
6. As written, the conclusion paragraph does not indicate well what the study found or how it extends the literature. The first sentence needs to be reworked—who is “their” referring to? Families were critical to “maintaining or improving “health and quality of life,” correct? Make succinct and specific mention to how the families were affected “beyond infection and physical symptoms.” What were the specific barriers that were exacerbated?

#### Minor Comments

1. “Other caregivers or their care recipient had been infected or were indeed infected during the interview.” This sounds like the interviewer infected them. They were experiencing COVID-19 at the time of the interview?
2. Typo in theme 4.3: “to the their.”
3. Avoid the numeric two in the quote in theme 5.3: “Mom had 2 that got COVID.” Suggested rework: “Mom had two [home assistants] that got COVID.”
4. Remove the hyphen from “frequently-mentioned.”

## Abbreviations

ADRD: Alzheimer disease and related dementias
